# Patients’ Experience With Evaluation by Both a Musculoskeletal Physician and Physical Therapist in the Same Digital Visit: Survey Study

**DOI:** 10.2196/66744

**Published:** 2025-03-03

**Authors:** Mary I O'Connor, Carolyn Chudy, Kaitlyn C Peters, Megan Ribaudo, Carrie McCulloch, Jared Aguilar, Trista Taylor, Ryan A Grant

**Affiliations:** 1 Vori Health, Inc, Nashville, TN, United States

**Keywords:** telemedicine, musculoskeletal care, patient satisfaction, multidisciplinary care, digital visit

## Abstract

**Background:**

Patients undergoing evaluation for musculoskeletal concerns are often seen by a physician and physical therapist in the in-person setting in a sequential manner. This process typically delays the onset of nonoperative care, inclusive of physical therapy, and creates the risk of inadequate clinical collaboration between physician and physical therapist. To address these issues, we designed a novel initial patient evaluation to a group visit in which both a specialty-trained musculoskeletal physician and physical therapist simultaneously evaluate a patient together in the digital encounter.

**Objective:**

The aim of the study is to gain insights from patients on their experience with this innovative digital simultaneous musculoskeletal medical doctor and physical therapist (MD+PT) visit format for the initial evaluation of musculoskeletal concerns.

**Methods:**

An electronic 7-question survey was sent to 750 patients who completed an MD+PT visit asking them to comment on prior musculoskeletal evaluations and their experience with the MD+PT format.

**Results:**

In total, 195 (26%) patients responded to the survey with the frequent body regions of diagnosis being lumbar spine (n=65), knee (n=32), shoulder (n=21), cervical spine (n=20), hip (n=14), and hand (n=11). Most patients had prior musculoskeletal experience with a physician or nurse practitioner (171/195, 87.7%) or physical therapist (148/195, 75.9%) with nearly all such encounters in the in-person setting (161/171,94.2% for physician or nurse practitioner and 144/148, 97.3% for physical therapy). Only 3.1% (6/193) of patients reported seeing both a physician and physical therapist during the same in-person visit. Patients rated the simultaneous MD+PT visit very favorably: this type of digital evaluation saved them time (179/192, 93.2%) and permitted them to promptly start their treatment plan (174/192, 90.6%). Overall, 87.5% (168/192) rated the MD+PT visit as enjoyable, and 92.2% (177/192) responded that it increased their confidence with understanding their medical condition and how to start treating it.

**Conclusions:**

Our early experience with the evaluation of patients with musculoskeletal conditions by both a specialty-trained musculoskeletal physician and physical therapist simultaneously in the same digital visit resulted in patients reporting a very positive experience with high satisfaction, engagement, and confidence in understanding their diagnosis and how to start treating it.

## Introduction

Musculoskeletal disorders are a leading cause of disability and health care expenditure worldwide [1]. Inappropriate care exacerbates the cost of musculoskeletal care as seen, for example, with high-cost spine [2] and total knee replacement [3] surgeries. Efficient and effective evidence-based evaluation of musculoskeletal disorders is critical to ensuring patient-centric care, delivering quality patient outcomes, and lowering health care costs. The type of care a patient with a musculoskeletal concern seeks and the way that care is delivered—especially at the onset of a care journey—can impact the risk of inappropriate and expensive imaging [4-6], procedures [7,8], and surgery [2,3,6]. In other words, what type of clinician a patient initially starts with influences the care path a patient will receive.

Most patients with musculoskeletal concerns first seek care from their primary care clinician [9]; however, many primary care clinicians lack fundamental competency in the musculoskeletal examination and do not feel comfortable evaluating, diagnosing, and treating musculoskeletal conditions [10]. In many states, patients may now directly see a physical therapist without a physician referral, via direct access physical therapy, due to the expansion of the scope of practice for physical therapists [11]. From either primary care or physical therapy, many patients are subsequently referred to an orthopedic or spine surgeon, or in the minority of cases a specialty nonoperative musculoskeletal physician (eg, physical medicine and rehabilitation or sports medicine physician), collectively referred to as specialty musculoskeletal physician. Creation of a comprehensive treatment plan for these patients will be dependent upon the completion of a specialty musculoskeletal physician’s clinical assessment, which often is delayed given the time required for separate physician and physical therapist appointments, and potentially by discordance in evaluations and advice by these 2 clinicians.

For many patients, musculoskeletal care can be effectively delivered through telemedicine. Digital delivery of medical services can be very convenient for patients, particularly for those with challenges to access in-person clinicians [12]. Physical examination of the patient with a musculoskeletal condition can be readily performed in the digital space [13]. The adequacy of that digital physical examination is supported by research showing good to very good concordance of the diagnosis of the patient with a musculoskeletal condition between the digital and in-person clinician [13]. Moreover, the concordance of treatment plans for patients with musculoskeletal conditions created in these 2 practice settings was “probably good to excellent” [14]. Finally, a meta-analysis of 35 systematic reviews assessing the use of telemedicine versus in-person care for musculoskeletal conditions showed telemedicine to be beneficial to or equal in patient-reported outcomes measures and objective measures, including physical function, and to have notably lower costs. Furthermore, in the small number of studies in the review reporting on patient experience measures, telemedicine was equivalent to in-person care [15].

We developed an initial clinical digital visit structure to address these delays in treatment created by the traditional sequential clinical visits between physician and physical therapist, streamline the patient experience, and advance evidence-based patient care. In this model, patients see a nonoperative musculoskeletal specialty physician (or a musculoskeletal nurse practitioner) and a physical therapist together—literally at the same time—during a simultaneous digital medical visit via our telehealth platform [16]. To our knowledge, such a simultaneous evaluation, which we will abbreviate as an “MD+PT” (musculoskeletal medical doctor and physical therapist) digital evaluation, has not been previously systematically implemented in either the in-person or digital care environment. To gain insights into the patient experience with this simultaneous patient-centric model, we created a descriptive survey to better understand the experience from the patients’ point of view.

## Methods

### Ethical Considerations

Our study, including the consent language and questionnaire, was reviewed by The Institute for Evaluation and Research Institutional Review Board and approved as exempt. Patients who completed an MD+PT digital evaluation were sent an email asking them to voluntarily consent to the research study and complete a 7-question survey about their appointment experience with the MD+PT evaluation (Textbox 1). All patients were 18 years or older of age. The email informed patients that the purpose of the study was to examine the impact of enabling patients to see a medical doctor or nurse practitioner and physical therapist together in the same digital appointment. Patients were provided no compensation for completing the survey. Those who elected to participate were informed that they could withdraw from the study at any time and skip questions if they chose to do so. The consent informed patients that their survey results would be confidential, shared with our researchers and not their medical team. Participants could answer the questionnaire in an anonymous manner and complete it only once. The questions could not be answered until participants consented to participate in the study. Data integrity was protected with access only provided to the researchers conducting the data analysis.

Textbox 1. **The 7-question survey used in this study of 195 patients who completed an evaluation with both a nonoperative musculoskeletal physician and physical therapist during the same digital encounter.**

1. Before coming to Vori Health, have you ever seen a medical doctor (or nurse practitioner or physician assistant) for back, muscle, or joint pain?

 - Yes, no

 If yes, were any of these visits telehealth appointments? Yes/no

2. Before coming to Vori Health, have you ever seen a physical therapist for back, muscle, or joint pain?

 - Yes, no

 If yes, were any of these visits telehealth appointments? Yes/no

3. Before coming to Vori Health, have you ever been seen by both a medical doctor/nurse practitioner and physical therapist together in the same appointment for back, muscle, or joint pain?

 - Yes, no

 If yes, were any of these visits telehealth appointments? Yes/no

The next few questions will ask you about your experience at Vori Health seeing a medical doctor or nurse practitioner and physical therapist together in the same appointment.

4. Seeing a medical doctor/nurse practitioner and physical therapist together in the same appointment saved me time.

 - Strongly agree, agree, neutral, disagree, strongly disagree

5. Seeing a medical doctor/nurse practitioner and physical therapist together in the same appointment increased my confidence in understanding my medical condition and how to start treating it.

 - Strongly agree, agree, neutral, disagree, strongly disagree

6. Seeing a medical doctor/nurse practitioner and physical therapist together in the same appointment helped me to start my treatment plan faster compared to if I had to see the doctor and physical therapist in different appointments.

 - Strongly agree, agree, neutral, disagree, strongly disagree

7. Seeing a medical doctor/nurse practitioner and physical therapist together in the same appointment made my care experience more enjoyable.

 - Strongly agree, agree, neutral, disagree, strongly disagree

 If disagree, or strongly disagree, show question below:

 Please indicate why the appointment was not enjoyable for you (choose all that apply):

  o The experience felt too complicated.

  o I did not feel I had enough time with each clinician.

  o I did not feel comfortable sharing confidential medical information with anyone other than my medical doctor.

  o Other (free text):

### Questionnaire

The questionnaire sought patients’ prior experience with musculoskeletal care, both in the in-person and telemedicine settings. Patients were asked if they had ever been seen by both a medical doctor (or nurse practitioner or physician associate) and physical therapist in the same appointment. Additional questions were asked related to patients’ experiences with their MD+PT digital evaluation. To recruit patients to the study, an email survey was sent to all patients who completed the MD+PT evaluation until we had 200 patients who consented to participate in the study. There were no other inclusion or exclusion criteria.

### The MD+PT Clinical Evaluation and Care Plan

The MD+PT digital evaluation was performed by a specialty musculoskeletal physician (typically, physical medicine and rehabilitation), or for a smaller number of patients by an experienced musculoskeletal nurse practitioner, and a physical therapist who had a minimum of 5 years of musculoskeletal experience. Each integrated visit was 40 minutes in duration. The physician or nurse practitioner, physical therapist, and patient were each in separate physical locations and logged into the same videoconferencing link to be together in the digital space for the evaluation. The history and digital physical examination were performed by a combination of physician or nurse practitioner and physical therapist. Each clinician could ask additional questions to the patient to provide more details to the medical history. The physical examination was completely digital and based on published guidelines [13]. Both the physician or nurse practitioner and physical therapist could instruct the patient to perform various movements and tests to complete the physical examination to the satisfaction of both clinicians. Based on the available evidence, the physician or nurse practitioner then rendered a medical diagnosis, and the physical therapist provided a functional diagnosis. If outside records, inclusive of previous imaging, were not available to the clinicians at the time of this initial visit, then the physician or nurse practitioner would request the outside records and imaging for subsequent review.

At the completion of this visit, patients were immediately started on an evidence-based biopsychosocial personalized care plan that may include evidence-based prescriptions, imaging, laboratory studies, and a personalized home exercise program facilitated by computer vision motion-tracking technology. Patients were subsequently scheduled for follow-up digital physical therapy care and, if applicable, dedicated health coaching and registered dietitian care, given the overlap of modifiable risk factors with musculoskeletal conditions (eg, obesity). Follow-up physician or nurse practitioner visits were also scheduled as clinically appropriate.

## Results

### Overview

Surveys were sent to a total of 750 patients, with 200 patients consenting to the study. In total, 5 patients consented but did not answer any questions on the survey resulting in 195 responses, a 26% survey response rate. Surveys were fully answered by 192 patients and incompletely answered by 3 patients who each did not complete 1‐2 questions. Patients who responded to the survey had a variety of diagnoses involving the following body regions: lumbar spine (n=65), knee (n=32), shoulder (n=21), cervical spine (n=20), hip (n=14), hand (n=11), ankle (n=8), elbow or wrist (n=6), thoracic spine (n=5), and other and nonspecific body regions (n=13).

### Prior Musculoskeletal Clinical Experience

Patients were asked about their prior experience with the evaluation of back, muscle, or joint pain. In total, 171 of 195 (87.7%) patients had previously seen a physician or nurse practitioner, with 161 of 171 (94.2%) of these encounters occurring in the in-person setting. A total of 148 of 195 (75.9%) patients had previously seen a physical therapist, with 144 of 148 (97.3%) of these encounters occurring in the in-person setting.

We asked patients about prior experience of being evaluated by both the physician (or nurse practitioner or physician assistant) and physical therapist in the same clinical visit for back, muscle, or joint pain. Only 3.1% (6/193) reported a prior visit that combined a physician and physical therapist with 1 patient commenting that this occurred during a hospital stay, with no patient reporting the encounter as digital. We concluded that our cohort had essentially no experience with simultaneous musculoskeletal clinical visits, particularly in the digital setting.

### MD+PT Digital Experience

The remainder of our survey questions focused on the experience of the patient with the MD+PT digital evaluation. Table 1 shows that 93.2% (179/192) agreed that this appointment format saved them time (strongly agreed: 162/192, 84.4% and somewhat agreed: 17/192, 8.9%).

**Table 1.** Survey responses (n=192) to question asking patients whether they felt the MD+PT digital evaluation saved them time.

Response	Participant count, n
Strongly agree	162
Somewhat agree	17
Neither agree nor disagree	11
Somewhat disagree	0
Strongly disagree	2

When patients were asked if the MD+PT digital evaluation allowed them to start their treatment plan faster than if they had seen a physician and physical therapist in separate appointments, 90.6% (174/192) of patients responded affirmatively (strongly agreed: 155/192, 80.7% and somewhat agreed: 19/192, 9.9%; Table 2).

**Table 2.** Survey responses (n=192) to question asking patient whether the MD+PT digital evaluation helped them start a treatment plan faster compared to seeing a doctor and physical therapist in separate appointments.

Response	Participant count, n
Strongly agree	155
Somewhat agree	19
Neither agree nor disagree	16
Somewhat disagree	1
Strongly disagree	1

Given our focus on the patient experience, the 2 most significant survey questions asked about patient enjoyment of their visit experience and confidence in medical decision-making. Overall, Table 3 shows that 87.5% (168/192) rated the visit as enjoyable (strongly agree: 135/192, 70.3% and somewhat agree: 33/192, 17.2%).

**Table 3.** Survey responses (n=192) to question asking patients whether the MD + PT digital evaluation made their patient care experience more enjoyable.

Response	Participant count, n
Strongly agree	135
Somewhat agree	33
Neither agree nor disagree	21
Somewhat disagree	1
Strongly disagree	2

The patients who responded as “somewhat disagreed” (n=1) or “strongly disagreed” (n=2) each provided a comment: (1) the practitioner and therapist were not very engaging, and I could not connect with them; (2) digital appointment diminished the transfer of information between the patient and provider; and (3) in-person health care is so very important, especially the first visit.

When asked if MD+PT digital evaluation increased their confidence with understanding their medical condition and how to start treating it, Table 4 illustrates that 92.2% (177/192) positively responded (strongly agree: 137/192, 71.4% and somewhat agree: 40/192, 20.8%).

**Table 4.** Survey responses (n=192) to question asking patients whether the MD+PT digital evaluation increased their confidence in understanding their medical condition and how to start treating it.

Response	Participant count, n
Strongly agree	137
Somewhat agree	40
Neither agree nor disagree	12
Somewhat disagree	1
Strongly disagree	2

## Discussion

### Overview

With 1 in 2 US adults estimated to experience a musculoskeletal disorder [9], improving musculoskeletal care must be a national, if not global, priority. We innovated our digital initial clinical evaluation to bring both the physician or nurse practitioner and physical therapist together simultaneously with the patient for the duration of the telemedicine evaluation. More specifically, both clinicians were together with the patient for the entirety of the first digital visit. This allows the patient to provide 1 history and the clinicians to collaboratively perform 1 digital physical examination. The physician or nurse practitioner renders a medical diagnosis, and the physical therapist provides a functional diagnosis, with the opportunity for collaboration between both clinicians during the visit to drive clinician concordance. The resultant treatment plan is aligned with the patient’s values and goals (ie, “What matters to me”) [17], and the patient can ask clarifying questions to both clinicians. Our clinicians subjectively commented that this integrated, combined visit was a more satisfying and collaborative experience for them.

### Principal Findings

Our survey results showed a very high level of positive responses from patients. Patients felt that this visit format saved them time (179/192, 93.2%), allowed them to start their treatment plan faster (174/192, 90.6%), and was enjoyable (168/192, 87.5%). Moreover, 92.2% (177/192) of patients responded that the visit increased their confidence with understanding their medical condition and how to start treating it. Improved patient understanding of their medical condition and associated treatment positively affects health outcomes [18]. Effective patient education supports an atmosphere of trust between patient and clinician, empowers patients to participate in their own health care, and supports informed decision-making [19].

We postulate that the high patient confidence in understanding their medical condition and how to start treating it was influenced by our MD+PT visit format. Our model was structured to create diagnostic and therapeutic agreements between clinicians. The model also provided patients with the benefit, real or perceived, of having both a musculoskeletal physician or nurse practitioner and physical therapist together with them in the clinical encounter. While direct access physical therapy has been shown to be a safe, less expensive, and effective model for triage of patients with musculoskeletal conditions compared to a required referral from a physician [20], physical therapists cannot order imaging, prescribe medications, or render a medical diagnosis in the United States. Patients may have a perception of a more complete evaluation when the physician (or nurse practitioner) is evaluating them, even if imaging or prescription medications are not ordered. Patients may also have a higher level of trust when a physician or nurse practitioner is creating the treatment plan with the physical therapist, particularly for patients with complex conditions or comorbidities. Future research comparing the experience of patients being evaluated by a physical therapist versus evaluation by both a physician or nurse practitioner and physical therapist may provide data on the potential value (or not) of the MD+PT visit.

Research on concordance [21] in diagnosis between physicians and physical therapists shows varied results. In a systemic review of 19 studies involving 1745 patients and a total of 35 advanced practice physical therapists (APPTs), Lafrance et al [22] showed concordance between APPTs and physicians for musculoskeletal disorders to be “probably good to very good for diagnosis” (κ coefficient [21] 0.76) and “good to very good for surgical triage” (κ coefficient 0.71). All studies in this systemic review were conducted outside the United States, and in 5 studies, APPTs had the authority to order diagnostic imaging studies, including magnetic resonance imaging [22]. The ability of some APPTs in this study to order diagnostic imaging limits the application of these results to concordance between a physical therapist and a physician in the United States.

In other research, Madsen et al [23] reported on 69 patients independently evaluated twice on the same day by an orthopedic surgeon and an extended scope physical therapist. Agreement between the surgeon and physical therapist was 62% on the primary diagnosis and 79% on a combination of diagnoses [23]. In 2 small studies, the κ coefficient between APPTs and musculoskeletal surgeons for referral for surgical treatment was 0.46 (shoulder disorders) [24] and 0.69 (lumbar spine pain) [25], suggesting an opportunity for improvement. The experience of the physical therapist is a variable that may influence these outcomes: in a study of 105 patients with carpal tunnel syndrome, physical therapists with advanced experience had 100% agreement with surgeons, whereas early-career physical therapists only had fair agreement [26]. Depressive symptoms were also shown in one study of knee disorders to decrease diagnostic concordance between physical therapists and medical musculoskeletal physicians [27]. While all our clinicians have a least 5 years of musculoskeletal clinical experience, our MD+PT model may mitigate diagnostic discordance when the clinical experience of one of the clinicians is more limited.

We believe that concordance in the treatment plan in our MD+PT model was facilitated by direct and immediate communication between clinicians. In a survey of interprofessional communication between Canadian orthopedic surgeons and physical therapists, 65.6% of clinicians in stand-alone practices report a negative view of communication compared to 48.4% of those in collaborative practice. This same study also found that orthopedic surgeons and physical therapists did not agree on the clinical information that should be shared regarding postoperative rehabilitation [28]. In a study of 600 survey responses of physical therapists treating patients who underwent rotator cuff repair, 33% of physical therapists reported receiving copies of the operative report in <25% of patients, and 16% reported not receiving an operative note >50% of the time. With postoperative therapy protocols based on the size of the rotator cuff tear and the complexity of the repair, this lack of communication could negatively impact clinical care [29].

We also highlight the importance of patients being able to promptly begin their treatment plan. In the traditional model, the time to diagnosis and treatment of a patient often depends upon the sequencing of physician and physical therapist evaluations as well as the potential need for insurance authorization for care, with frequent delays in care. While in many states, patients can access physical therapy (direct PT) for initial treatment with no delay in beginning the physical therapy plan of care, one small study found that 25.6% of patients who began physical therapy and were subsequently referred to a physician had their physician visit at a range of 0.5 to 10 weeks following the initial physical therapy evaluation [30]. We were unable to find data on the length of time for a patient to see the physical therapist after referral from the physician, which is likely dependent on many factors including patient insurance and the need for prior authorization. Delaying the onset of treatment may lengthen the recovery time and negatively impact clinical outcomes [31,32]. Finally, 2 separate clinical visits can result in additional expenses to patients related to travel costs, time lost from work, and disruption of normal activities.

Finally, a very high percentage (168/192, 87.5%) of patients found the MD+PT visit enjoyable. We chose to ask this question as an attempt to gauge the emotional experience of the patient with 1 simple question. We believe that taking evidence-based care to the next level of effectiveness also requires delivering a better patient experience. We are not aware of others using this question to gain insights into the patient experience. The current Consumer Assessment of Healthcare Providers and Systems (CAPHS) survey, a standard instrument for measuring the patient experience, asks patients about various aspects of their experience including whether their visit started on time and if the clinician showed them respect, listened carefully, and gave them enough time. CAPHS asks patients to rate their visit on a scale of 0 (worse visit possible) to 10 (best visit possible) [33]. In our opinion, CAPHS is a very valuable tool but does not query the emotional response of the patient to the encounter. While we recognize “enjoyable” may never be a realistic experience for patients with very serious medical issues, we challenge the health care system to explore this, and similar, questions that assess the emotional response of the patient. We believe that the more enjoyable the clinical experience is for patients, the higher their engagement will be, which will translate into improved clinical outcomes.

### Limitations

Our research has several limitations. The main limitation was our survey response rate of 26% (N=195) raising the potential for nonresponder selection bias. Consensus as to an acceptable survey response rate in medical research is lacking [34,35]. Studies show a range of patient response rates to email surveys. In a global overview paper of surgical patients, email studies gave an average response rate of 68%, although the United States was noted to have a lower overall response rate of 64.2% [33]. In a primary care practice, the response rate to a postvisit email survey was 60.9%. However, only 45.15% of patients who initially consented to completing a paper waiting room survey agreed to receive an email survey [36].

While these response rates are higher than ours, they may not be directly comparable. Patients who have undergone a surgical procedure may be more willing to participate in a research study compared to those who had a telemedicine visit. In the primary care practice study cited in the preceding paragraph, the response rate to an email survey was 27.7% (60.9% of the 45.15% of patients who consented to an email survey), which is comparable to our response rate of 26% (N=195). In our study, we did not ask patients for consent to be surveyed, rather we sent the email survey to all eligible patients until 200 patients consented to the study.

In studies with methodology more aligned with ours, our survey response rate is favorable. A 2016 primary care study showed a 19.8% response rate to a 6-question email survey to patients on access to care and patient centeredness [37]. Another publication in 2018 reported 28% of patients opening an email containing a patient experience survey, with 80% completing the survey [38]. In a 2024 poster on patient satisfaction and outcomes, an email survey of patients having a genetic information session for kidney disease via telephone had a response rate of 12.1%, although the survey was much longer at 47 questions [39].

We did not conduct a corresponding survey of our clinicians using this model to potentially identify episodes of initial discordance in clinical assessment and treatment plan (and how these were resolved). All patients received a personalized, evidence-based, biopsychosocial treatment plan supported by the physician or nurse practitioner and physical therapist at the completion of the initial visit. Health coaches and registered dietitians were brought into the clinical team to support the patient as appropriate. We also did not survey our clinicians on their perception of whether communication between clinicians was facilitated with this model or whether the clinicians found this model to be enjoyable for them. We did not correlate the results of this survey with clinical outcomes or health care use; these are areas we plan to study in the future.

Patient’s perception of the quality of care provided by each clinician, or the quality of care provided by the MD+PT visit, was not studied and is an area for potential future research. However, given that patients reported high confidence in their medical plan with the MD+PT visit, we postulate that this is a surrogate for patients’ perceptions of a higher quality of care. In the literature, one study found that patients with osteoarthritis reported that physical therapists provided better information about how to take care of their condition, while surgeons provided patients with greater involvement in the decision-making process and facilitated a greater degree of expectations being met [40]. When compared to primary care clinicians, another study found that patient perception of physical therapy–led care was superior in all quality aspects evaluated [41].

Our model requires the resources of both a physician or nurse practitioner and physical therapist in the initial evaluation. Direct access physical therapy has been shown to be clinically effective and resource-sensitive, as many patients with musculoskeletal conditions will not require a physician or nurse practitioner assessment [11]. The scope of our study did not support any type of cost analysis of our model versus direct access physical therapy. We believe that patients with low-complexity musculoskeletal issues can be effectively evaluated in a physical therapy first model and those with high complexity are better evaluated in our MD+PT model.

Finally, our study did not directly address whether health literacy was improved in our patients. Health literacy is defined as “the degree to which individuals have the ability to find, understand, and use information and services to inform health-related decisions and actions for themselves and others” [42]. We speculate that patients having increased confidence in their understanding of their condition and how to start treating it may have greater health literacy. As annual health care costs of individuals with low health literacy are 4 times higher than those of higher health literacy individuals, and patients with higher health literacy are more likely to adhere to treatment recommendations [43], we will continue our research to identify the potential impact of our model on health literacy.

### Conclusions

We found our innovative model of a musculoskeletal physician or nurse practitioner and physical therapist evaluating the patient with a musculoskeletal condition together (at the same time during the initial telemedicine visit) resulted in a high degree of positive patient feedback. Overwhelmingly, patients reported that this visit model saved them time, allowed them to start their treatment plan faster, was enjoyable, and increased their confidence in understanding their medical condition and how to start treating it. We will continue to seek additional ways to innovate patient-centric care that enhances the patient experience, clinical outcomes, and appropriate health care use.

## Abbreviations

APPT: advanced practice physical therapist
CAPHS: Consumer Assessment of Healthcare Providers and Systems
MD+PT: musculoskeletal medical doctor and physical therapist
